# Effects of Taraxaci Herba (Dandelion) on Testosterone Propionate-Induced Benign Prostatic Hyperplasia in Rats

**DOI:** 10.3390/nu16081189

**Published:** 2024-04-17

**Authors:** Seong Min Lee, Sang Mok Lee, Jungbin Song

**Affiliations:** Department of Herbal Pharmacology, College of Korean Medicine, Kyung Hee University, 26 Kyungheedae-ro, Dongdaemun-gu, Seoul 02447, Republic of Korea

**Keywords:** Taraxaci Herba, testosterone propionate, benign prostatic hyperplasia, testosterone, dihydrotestosterone

## Abstract

Benign prostatic hyperplasia (BPH) is the non-malignant enlargement of the prostate, associated with lower urinary tract symptoms (LUTSs). Taraxaci Herba (TH), commonly known as dandelion, has traditionally been utilized in East Asia to treat symptoms related to LUTSs. Based on this traditional use, our study aimed to explore the inhibitory effects of TH on BPH progression using a testosterone propionate-induced rat model. To induce BPH, male Sprague Dawley rats were castrated and injected subcutaneously with testosterone propionate (3 mg/kg/day) for 28 days. Concurrently, TH extract was administered orally at doses of 100 and 300 mg/kg/day throughout the four-week period of testosterone propionate injections. The TH extract significantly reduced both the absolute and relative weights of the prostate, along with histopathological changes in the gland. Moreover, it lowered serum levels of testosterone and dihydrotestosterone and reduced the expression of the androgen receptor in the prostate. Additionally, the TH extract modulated the protein expressions of Bax and Bcl-2, which are key regulators of apoptosis in prostate cells. Collectively, our findings suggest that TH inhibits BPH development partially by modulating androgen signaling and inducing apoptosis within the prostate.

## 1. Introduction

Benign prostatic hyperplasia (BPH) refers to the non-malignant enlargement of the prostate, which is associated with lower urinary tract symptoms (LUTSs). More than 80% of men over the age of 80 experience LUTSs/BPH, encompassing symptoms such as nocturia, a weak urinary stream, straining to void, and an intermittent stream [1,2]. The increased proliferation of epithelial and stromal cells in the prostate’s transition zone leads to abnormal prostatic hyperplasia [3]. Although the exact etiology of BPH remains elusive, contributing mechanisms primarily include an age-related imbalance of androgens and estrogens, chronic inflammation, and an increase in prostatic cellular growth factors [4,5,6]. Among these, the dysregulation of androgens, especially testosterone and dihydrotestosterone (DHT), is particularly significant. Testosterone is converted into the more potent DHT by 5α-reductase in the prostate, playing a crucial role in BPH progression. Both testosterone and DHT bind to the androgen receptor (AR) within prostate cells, with DHT exhibiting a higher affinity for the AR compared to testosterone, making it a more potent androgen [7]. The binding of androgens to AR stimulates the expression of growth factors and promotes the proliferation of prostate cells [8].

Currently, alpha-blockers, 5α-reductase inhibitors, and their combination are used for the pharmacotherapy of BPH. The use of PDE-5 inhibitors also represents a potential treatment option [9]. Alpha-blockers relax smooth muscle tone, thereby alleviating LUTSs, but they cannot prevent BPH progression. Tamsulosin, a commonly prescribed uroselective α1_A_-adrenoceptor antagonist, has been reported to induce ejaculation-related side effects in 8.4–18.1% of cases, depending on the dosage [10,11]. 5α-reductase inhibitors, which inhibit the conversion of testosterone to DHT, lead to libido loss in 3 to 5% of men and erectile dysfunction in 5 to 16% of men [12,13]. Concerns about these sexual side effects have led many BPH patients to choose herbal treatments as alternative options [14].

The genus Taraxacum, commonly known as dandelions, has been utilized as a food source and medicinal herb for centuries across Asia, North America, and Europe. The Korean Herbal Pharmacopoeia recognizes the whole plants of *Taraxacum platycarpum*, *T. officinale*, *T. mongolicum*, and *T. coreanum* (of the Compositae family) as Taraxaci Herba (TH). In East Asia, TH has been employed to treat inflammatory disorders such as infectious skin diseases and appendicitis since its documentation in the *Tang Materia Medica* (also known as the *Newly Revised Materia Medica*), the world’s first pharmacopoeia, published during the Tang Dynasty in China in 659 AD [15]. Research focusing on the pharmacology of the Taraxacum genus has primarily been preclinical and has revealed that TH possesses pharmacological benefits, including antioxidant, anti-obesity, anticancer, diuretic, anti-inflammatory, and immunoprotective effects [16,17]. These pharmacological effects are attributed to its bioactive chemical components, which include sesquiterpenoid lactones (e.g., taraxacoside, taraxinic acid, taraxacolide-*O*-β-glucopyranoside), phenolic acids (e.g., chlorogenic acid, caffeic acid, chicoric acid), flavonoids (e.g., chrysoeriol, quercetin, luteolin-7-glucoside), triterpenoids (e.g., α-amyrin), and sterols (e.g., taraxasterol, β-sitosterol, stigmasterol) [18,19,20].

TH has been used as a folk remedy for treating LUTSs in Western and Eastern Asia, as well as in Europe [20,21]. Especially in Korea and China, TH has been traditionally used to treat stranguria, which is a predominant symptom in patients with BPH [22,23]. Given its traditional use, we anticipated potential inhibitory effects of TH on BPH. Although a recent study has suggested an inhibitory effect on BPH from a formulation mixture of *T. officinale*, *Nelumbo nucifera*, and *Cuscuta australis* [24], the efficacy of TH alone in treating BPH has not been previously reported. In the current study, we investigated the inhibitory effects of TH on BPH using a testosterone propionate (TP)-induced rat model and explored the possible underlying mechanisms.

## 2. Materials and Methods

### 2.1. Preparation of TH Extract

Dried TH, produced in Gyeongsangbuk-do, Korea, was purchased from Dongwoodang pharmacy Co., Ltd. (Yeongcheon-si, Gyeongsangbuk-do, Republic of Korea), and its authenticity was confirmed by Prof. Hocheol Kim of Kyung Hee University. The voucher specimen was stored in the Herbarium of Dept. of Herbal Pharmacology, College of Korean Medicine, Kyung Hee University (No. 21111201). The dried plant material was reflux-extracted in 70% ethanol with a 1:20 solid-to-liquid ratio for 3 h at 80 °C and then was filtered through cellulose filter papers (Whatman™ grade 2 filter papers, Cytiva, Marlborough, MA, USA). The filtrate was concentrated at 45 °C using a rotary evaporator under reduced pressure and was subsequently dried in a freeze dryer (FDU-1200, EYELA, Tokyo, Japan), resulting in a powder with an extraction yield of 24.7%.

### 2.2. Animals

Male Sprague Dawley rats which were 10 weeks old and specific pathogen-free were purchased from Koatech (Pyeongtaek-si, Gyeonggi-do, Republic of Korea) and used in accordance with the guidelines of the Institutional Animal Care and Use Committee (IACUC) of Kyung Hee University. The animal study protocol was approved by the IACUC of Kyung Hee University prior to the experiment (Approval No. KHSASP-23-130, date of approval 12 April 2023). The rats were housed in an environment maintained at a temperature of 23 ± 1 °C with a humidity of 55–60% and a 12 h light–dark cycle. The rats received a standard chow diet and were allowed free access to distilled water throughout the study. Before the start of the experiment, it was planned to humanely terminate the experiment and exclude any rat from the analysis in the case of complications due to castration, abnormal clinical signs, or rapid weight loss of more than 20% compared to the rat’s normal body weight.

### 2.3. TP-Induced Benign Prostatic Hyperplasia Rat Model

Following a 1-week acclimatization period, a total of 41 rats were randomly assigned to one of five groups: sham (*n* = 6), control (*n* = 9), finasteride 1 mg/kg (*n* = 8), TH extract 100 mg/kg (*n* = 9), and TH extract 300 mg/kg (*n* = 9). The sample size was determined based on previous research [25,26]. Rats were allocated to each group using a stratified random sampling method based on body weight to ensure equal average weights across groups.

All rats, except the sham, were castrated under isoflurane anesthesia after group allocation. Castration was conducted to rule out the effects of endogenous testosterone [25]. The testicles, epididymis, and epididymal fat were removed via the abdominal approach as described in the previous literature [27]. After the surgery, the rats were kept under standard conditions for a week for recovery. BPH was induced in the castrated rats by subcutaneous injections of TP (3 mg/kg/day dissolved in olive oil) for 28 continuous days. The sham group received olive oil injections in the same regimen. Concurrently with TP injections, TH extract and finasteride (Proscar^®^, MSD, Seoul, Republic of Korea) were orally administered daily for 28 days, whereas the sham and BPH control groups were given distilled water following the same regimen.

After the dosing period, the rats were fasted overnight and sacrificed under isoflurane anesthesia. Blood samples were collected from the vena cava and the prostate tissues, including the ventral, dorsolateral, and anterior lobes, and were quickly isolated and weighed. The prostate index was calculated as the ratio of the prostate weight (in mg) to the fasting body weight of each rat (per 100 g) measured on the day of sacrifice. The right ventral lobes were fixed with 10% formalin for histological examination. The left ventral lobes were snap-frozen using liquid nitrogen for Western blot analysis.

### 2.4. Administration of TH Extract and Positive Drug

The TH extract was administered orally at doses of 100 and 300 mg/kg/day, while the positive drug, finasteride (Proscar^®^, MSD, Seoul, Republic of Korea), was administered orally at a dose of 1 mg/kg/day. Both the TH extract and finasteride were dissolved in distilled water and administered at a volume of 10 mL/kg body weight. Oral administration was carried out daily between 8:30 AM and 11:30 AM, and the order of treatments was randomized daily according to the ARRIVE guidelines [28].

According to the World Health Organization [29], the daily human dosage of TH is 9–12 g/day as dry material. Considering a yield of 24.7% for the TH extract and taking into account the difference in body surface area between humans and rats [30], this human dosage corresponds to an extract dosage of approximately 230–300 mg/kg/day in rats. These oral doses have been reported to be safe in both humans and animals [29,31,32]. Based on this rationale, we set the high dose at 300 mg/kg/day, and the low dose was determined to be one-third of this amount, at 100 mg/kg/day.

### 2.5. Histology

The fixed ventral prostate tissues were embedded in paraffin, sectioned into 4 μm thick slices, and stained using Harris’ Hematoxylin and Eosin method. The sections were then observed using an upright microscope (Eclipse Ci-L, Nikon, Tokyo, Japan). For histological evaluation, a previously developed scoring protocol [33,34] was employed under both low- (100×) and high-power magnifications (400×). Epithelial thickness was quantified using ImageJ software (ver. 1.54a, NIH, Bethesda, MD, USA) at 100× magnification, measuring five random sites for each rat and calculating the average value. The histological evaluation was conducted by the researcher blinded to the group allocation.

### 2.6. Determination of Serum Testosterone and DHT Concentrations

The whole blood was temporarily stored at 4 °C on the day of sacrifice, and the clotted blood was centrifuged at 4000 rpm and 4 °C for 10 min. The serum was then obtained and stored at −80 °C until analysis. Concentrations of testosterone and DHT in serum were measured using competitive inhibition enzyme-linked immunosorbent assay kits (Cat. No. CSB-E05100r and CSB-E07879r, Cusabio Technology, Houston, TX, USA). The procedure was carried out in accordance with the guidelines provided by the manufacturer.

### 2.7. Western Blot Analysis

Ventral prostatic tissue samples were homogenized in a RIPA lysis and extraction buffer (Thermo Scientific, Waltham, MA, USA), employing the Bead Ruptor Elite (OMNI International, Kennesaw, GA, USA). Following homogenization, samples were centrifuged at 13,000 rpm for 10 min at 4 °C. The supernatant was then transferred to a clean microcentrifuge tube for further analysis. The protein concentration within the supernatant was determined using a Pierce™ BCA protein assay kit (Thermo Scientific, Waltham, MA, USA). For the gel electrophoresis, a 2× Laemmli sample buffer (Bio-Rad Laboratories, Hercules, CA, USA) was used to prepare the loading sample, with 60 µg of protein loaded into each well of 4–20% Mini-PROTEAN^®^ TGX™ precast protein gels (Bio-Rad Laboratories). Electrophoresis was conducted at 200 V for 30 min, followed by protein transfer onto PVDF membranes at 100 V for 60 min. To block non-specific binding, the membranes were incubated in 5% skim milk in TBST at room temperature for 1 h. Then, the membranes underwent overnight incubation at 4 °C with primary antibodies targeting AR (1:200), Bcl-2 (1:1000), Bax (1:1000), and β-actin (1:500), supplied by Santa Cruz Biotechnology (Dallas, TX, USA). After removing the primary antibody solution, the membranes were washed four times, each for 5 min with TBST (Bio-Rad Laboratories). The secondary antibody incubation involved goat anti-mouse IgG (H+L) (Invitrogen, Waltham, MA, USA) at dilutions of 1:5000 for β-actin and Bcl-2 and 1:2000 for AR and Bax, conducted at room temperature for 1 h. Following four 5 min washes with TBST, protein bands were visualized using the WesternBright ECL HRP substrate (Advansta, San Jose, CA, USA). The chemiluminescence was captured using a ChemiDoc MP Imaging System (Bio-Rad Laboratories, Hercules, CA, USA). Protein expression levels were analyzed with Image Lab Software (ver. 6.1, Bio-Rad Laboratories, Hercules, CA, USA) and normalized to β-actin levels.

### 2.8. Statistical Analysis

Group differences were analyzed using an analysis of variance, followed by Dunnett’s multiple comparison test, employing GraphPad Prism software (ver. 8.4.2, GraphPad Software, Inc., San Diego, CA, USA). There were no data points excluded from the analysis. A *p*-value of less than 0.05 was considered statistically significant. Data are expressed as mean ± standard deviation (SD).

## 3. Results

### 3.1. Body Weight Changes

**Figure 1:**
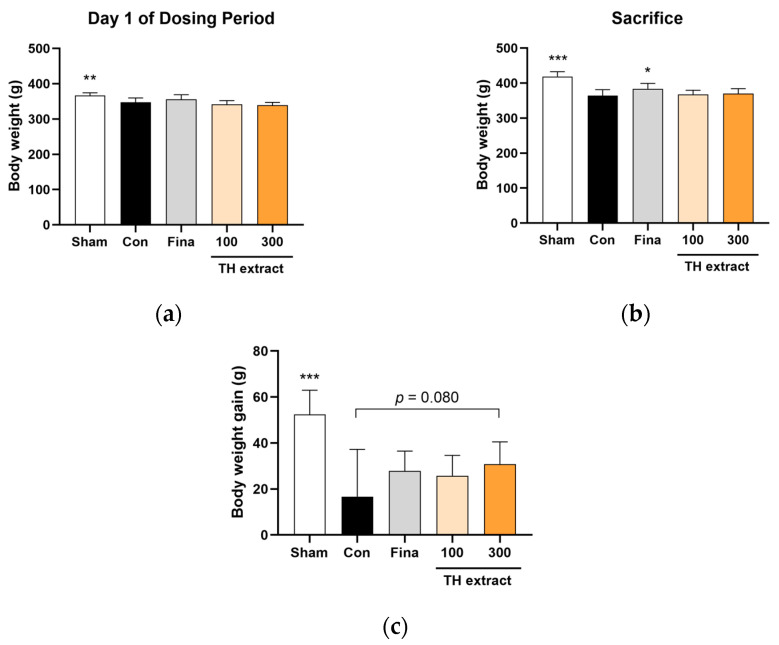
Effects of Taraxaci Herba (TH) extract on body weight changes in testosterone propionate injected rats. (**a**) Body weight on day 1 of the dosing period. (**b**) Fasting body weight at sacrifice. (**c**) Body weight gain during the 28-day dosing period. All values are presented as the mean ± SD (*n* = 6 for sham, 8 for fina, and 9 for the other groups). * *p* < 0.05, ** *p* < 0.01, and *** *p* < 0.001 vs. control group (Con). Fina, finasteride 1 mg/kg.

At the beginning of the dosing period, the body weights across the BPH groups (four groups excluding the sham) were not statistically different (Figure 1a). The administration of TP at levels exceeding physiological ranges has been reported to suppress body weight gain in castrated adult rats [35]. In accordance with this previous study, the body weight on the day of sacrifice and the body weight gain during the 28-day dosing period were significantly lower in the BPH control group compared to the sham group (Figure 1b,c). While the intergroup difference was not statistically significant, the administration of TH extract at 300 mg/kg exhibited a tendency to increase body weight gain compared to the control group (31.0 ± 9.6 vs. 16.7 ± 20.6 g, *p* = 0.080).

### 3.2. Prostate Weight and Prostate Index

**Figure 2:**
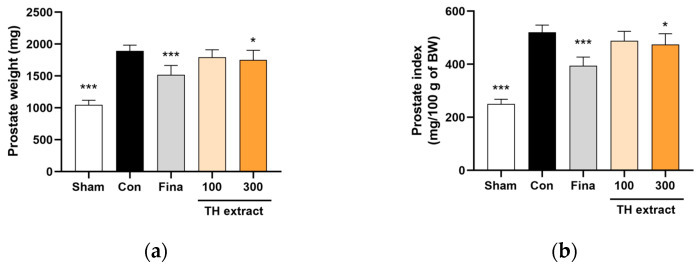
Effects of Taraxaci Herba (TH) extract on the prostate weight (**a**) and prostate index (**b**) of rats with testosterone propionate-induced benign prostatic hyperplasia. The prostate index was calculated as the prostate weight (mg) per 100 g of fasting body weight (BW) at sacrifice. All values are presented as the mean ± SD (*n* = 6 for sham, 8 for fina, and 9 for the other groups). * *p* < 0.05 and *** *p* < 0.001 vs. control group (Con). Fina, finasteride 1 mg/kg.

After 4 weeks of TP administration, the prostate weight in the control group exhibited an approximately 1.8-fold increase compared to the sham group (1893.7 ± 91.4 vs. 1047.5 ± 71.3 mg, *p* < 0.001; Figure 2a). The administration of TH extract at 300 mg/kg and finasteride at 1 mg/kg significantly reduced prostate weight increases by 22.6% (*p* < 0.05) and 37.9% (*p* < 0.001), respectively.

The prostate index, representing prostate weight relative to body weight, significantly increased approximately 2.1-fold in the control group compared to the sham group (520.9 ± 26.7 vs. 250.3 ± 18.1, *p* < 0.001; Figure 2b). The increase in prostate index was significantly inhibited by 21.3% with TH extract at 300 mg/kg (*p* < 0.05) and by 41.7% with finasteride at 1 mg/kg (*p* < 0.001).

### 3.3. H&E-Stained Prostate Tissues

**Figure 3:**
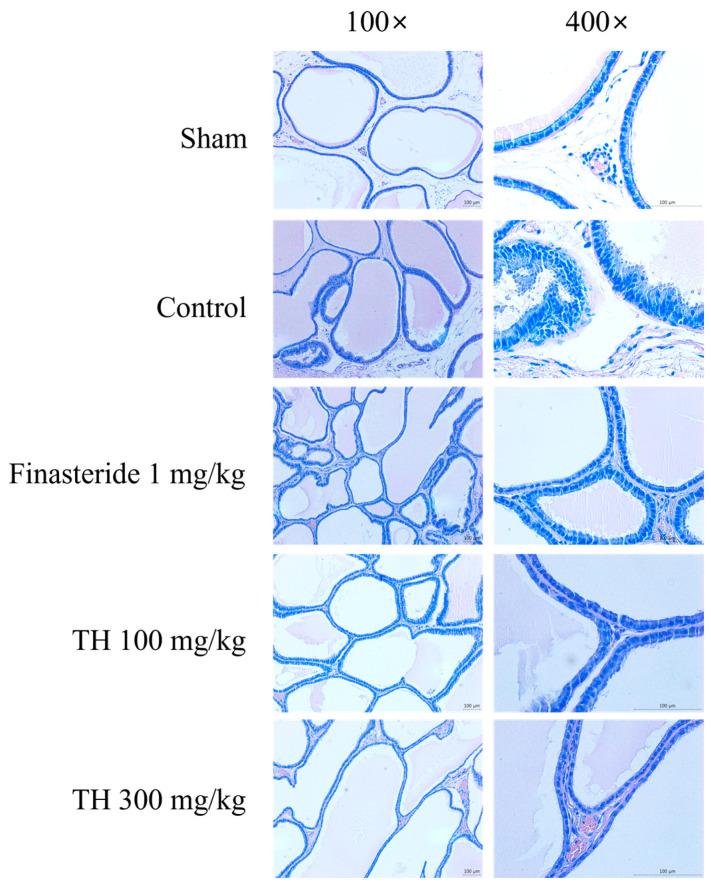
H&E staining of the rat ventral prostate shown at magnifications of 100× and 400×. Scale bar = 100 μm at both magnifications. TH, Taraxaci Herba extract.

Histological analysis using H&E staining was performed to assess the effects of TH extract on prostate hyperplasia. Figure 3 shows the morphological alterations in the ventral prostate of each group, observed at 100× and 400× magnifications. Normal cellular morphology was observed in the sham group, whereas the control group displayed increased glandular hyperplasia and reduced luminal space. The cellular structure in the group treated with finasteride was similar to that of the sham group. The administration of TH extract at both 100 and 300 mg/kg doses resulted in a reduction in glandular hyperplasia.

### 3.4. Histological Scores

Table 1 shows the histological scores, determined by observing alterations in the histology of the glandular epithelium and stroma under low-power (100×) and high-power (400×) magnification according to the previously developed scoring protocol [33,34]. This assessment encompassed various factors including the shape of luminal and acinar structures, the space between acini, stromal features, epithelial morphology, layering, outward growth, the distribution of lesions, and the shape and size of nuclei. The sham group, which showed no apparent lesions in the ventral prostate, had a total score of 21.67 ± 8.36. In contrast, the total score of the control group was 53.89 ± 5.23, which was significantly higher than that of the sham group (*p* < 0.001). TP in the control group resulted in histopathological alterations typical of benign prostatic hyperplasia, including irregular acinar structures, nuclear shape and size variation, a disrupted basement membrane, cellular layering, and budding. These pathological manifestations were mitigated following the administration of TH and finasteride. The treatment with TH extract at doses of 100 mg/kg and 300 mg/kg and finasteride at 1 mg/kg significantly reduced the total scores to 34.33 ± 17.70, 26.33 ± 14.31, and 22.00 ± 6.95, respectively, compared to the control group.

### 3.5. Epithelial Thickness of the Prostate

**Figure 4:**
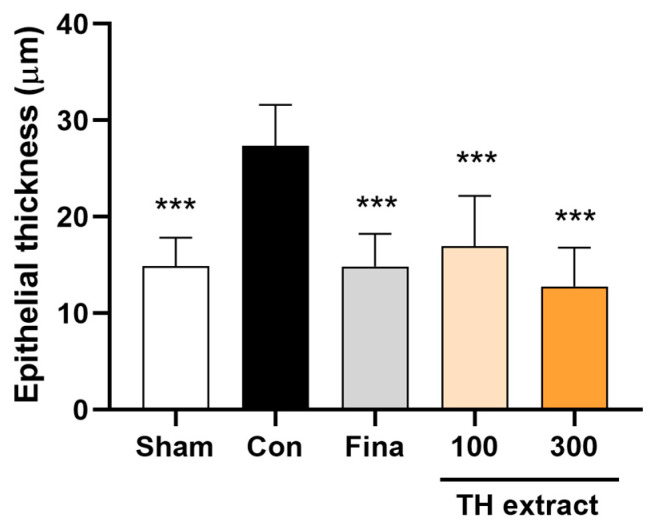
Epithelial thickness of the rat ventral prostate. All values are presented as the mean ± SD (*n* = 6 for sham, 8 for fina, and 9 for the other groups). *** *p* < 0.001 vs. control group. Fina, finasteride 1 mg/kg.

The control group exhibited a significant increase in the epithelial thickness of the ventral prostate compared to the sham group (27.3 ± 4.3 vs. 14.9 ± 3.0 µm, *p* < 0.001; Figure 4). The administration of TH extract at doses of 100 and 300 mg/kg and finasteride at 1 mg/kg significantly reduced the epithelial thickness compared to the control group (all *p* < 0.001).

### 3.6. Serum Testosterone and DHT Concentrations

**Figure 5:**
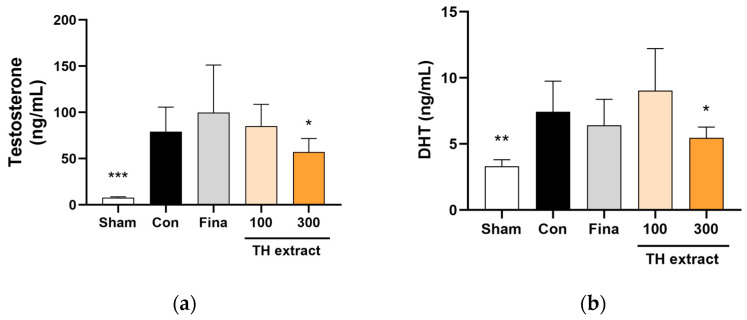
Effects of Taraxaci Herba (TH) extract on serum testosterone (**a**) and dihydrotestosterone (DHT; (**b**)) levels in rats with testosterone propionate-induced benign prostatic hyperplasia. All values are presented as the mean ± SD (*n* = 6 for sham, 8 for fina, and 9 for the other groups). * *p* < 0.05, ** *p* < 0.01, and *** *p* < 0.001 vs. control group. Fina, finasteride 1 mg/kg.

The serum testosterone levels were significantly higher in the control group compared to the sham group (Figure 5a). The TH 300 mg/kg group showed significantly lower serum testosterone levels compared to the control group (57.1 ± 14.7 vs. 79.3 ± 26.4 ng/mL, *p* < 0.05).

The serum DHT levels in the control group showed a significant increase of approximately 2.2-fold compared to the sham group (7.4 ± 2.3 vs. 3.3 ± 0.5 ng/mL, *p* < 0.01; Figure 5b). Treatment with 300 mg/kg of TH extract inhibited the increase in serum DHT levels by 48.3%, reaching 5.5 ± 0.8 ng/mL (*p* < 0.05 vs. control).

### 3.7. Protein Expressions of AR and Apoptosis-Related Biomarkers

**Figure 6:**
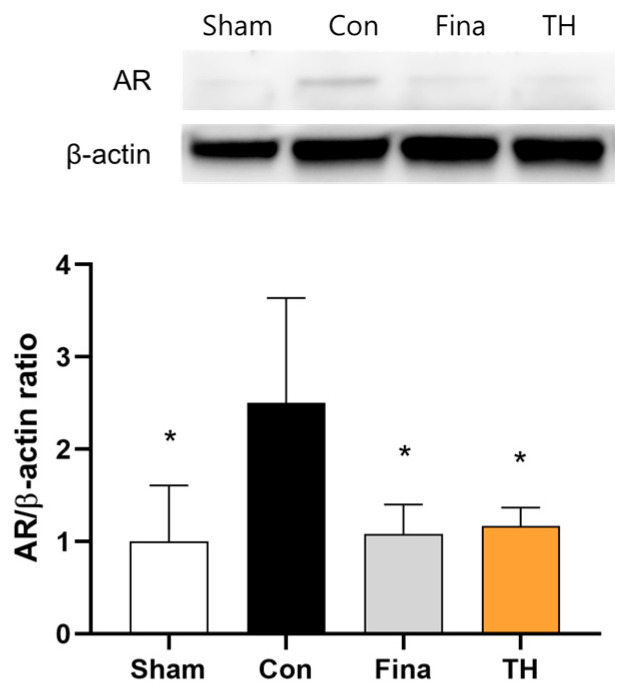
Effects of Taraxaci Herba (TH) extract on protein expressions of androgen receptor in the ventral prostate of rats injected with testosterone propionate. All values are presented as the mean ± SD. * *p* < 0.05 vs. control group. Fina, finasteride 1 mg/kg; TH, Taraxaci Herba extract 300 mg/kg.

Androgens, primarily testosterone and DHT, bind to ARs upon reaching prostate tissue. After we found that TH reduced serum androgen levels, we evaluated the effects of TH on the expression of the AR in ventral prostate tissue. The control group showed higher expression of AR protein in the ventral prostate compared to the sham group (Figure 6). This increased expression was reduced following the administration of TH extract (300 mg/kg) and finasteride (1 mg/kg).

**Figure 7:**
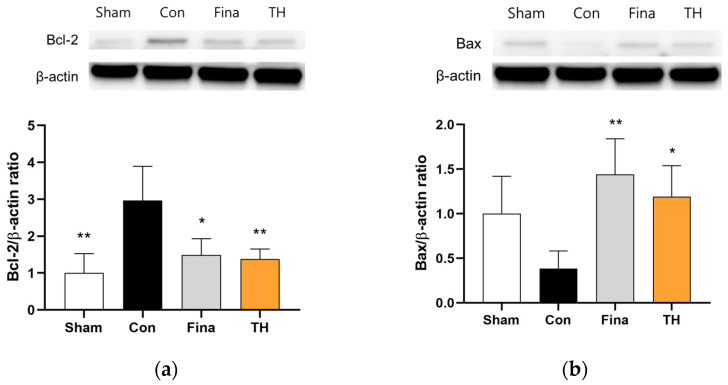
Effects of Taraxaci Herba (TH) extract on protein expressions of Bcl-2 (**a**) and Bax (**b**) in the ventral prostate of rats injected with testosterone propionate. All values are presented as the mean ± SD. * *p* < 0.05 and ** *p* < 0.01 vs. control group. Fina, finasteride 1 mg/kg; TH, Taraxaci Herba extract 300 mg/kg.

To determine the effects of TH on apoptosis signaling, we assessed the protein expression of anti-apoptotic Bcl-2 and pro-apoptotic Bax in the ventral prostate. TH extract reduced the expression of Bcl-2 and increased the expression of Bax compared to the control group (Figure 7).

## 4. Discussion

The present study investigated the inhibitory effects of TH on BPH development, using a TP-induced rat model of BPH. The findings revealed that the oral administration of TH significantly mitigated BPH progression, evidenced by reduced prostate weights and histopathological changes. Additionally, TH decreased serum androgen levels and modulated the expression of AR and genes associated with apoptosis.

The TP-induced BPH rat model used in this study is a well-established experimental model for studies on BPH [36]. There are several methods to induce a BPH model, including sex hormone, inflammation, urogenital sinus, and phenylephrine-induced models [37]. Of these, the sex hormone—predominantly androgen—induced rat model is the most established and commonly used method, yet this model is not suitable for nonhormonal therapy studies and employs high hormone doses that do not accurately mimic the actual disease state [25,37]. Typically, BPH in rats is induced through subcutaneous injections of TP, either following castration or without castration. Castration offers the advantage of eliminating the influence of endogenous testosterone [25]. Subcutaneously injected testosterone and its metabolite, DHT, bind to the AR in the prostate. Upon activation, the AR, a nuclear receptor, functions as a transcription factor of growth factors, consequently accelerating prostate stromal and epithelial cell proliferation, which leads to prostatic enlargement [38]. Increased prostate weight due to such proliferation is a surrogate marker of BPH progression [39]. In the present study, control rats had a significantly increased absolute and relative prostate weight compared with the sham group, indicating that BPH was well induced in this study. TH extract significantly reduced the absolute and relative weights of prostates in rats with TP-induced BPH compared to the control group. Decreased prostate weight after the administration of TH extract suggests that TH inhibits the hyperplastic progression of the prostate in TP-induced BPH rats.

Histopathological observations revealed that the oral administration of TH extract mitigated TP-induced hyperplasia in the ventral prostate. Testosterone administration to rats induces the irregular growth of acinar structures as well as stromal tissues in the prostate [33,40]. In particular, it leads to notable histopathological alterations in the glandular epithelium of the ventral prostate, including changes in cell shape (cylindric) and a papillary-like configuration [33], which are in line with our results. Thus, epithelial thickness is considered as a marker for BPH [40]. In this study, discrete clusters of epithelial cells were also identified outside the acinar structures. Moreover, disruptions and thickening of the basement membrane were observed. These histopathological changes induced by TP align with previous studies [41,42] and were alleviated by TH, as evidenced by significantly lower histological scores and epithelial thickness compared to the control group. Alongside the decreased prostate weight, these histopathological results suggest that TH possesses an inhibitory effect on benign prostatic hyperplasia.

Androgens and the activation of AR pathways have been linked to the development and maintenance of BPH. Testosterone, the primary androgen predominantly secreted by the testes, is converted to DHT by 5α-reductase enzymes upon reaching the prostate. Within prostate cells, DHT binds to the AR with two- to five-fold higher affinity and increases AR signaling tenfold compared to testosterone [8,43]. The androgen receptor, a member of the steroid and nuclear receptor family, translocates to the nucleus upon androgen binding and regulates the expression of genes related to apoptosis and proliferation. In this study, TP administration significantly increased the serum levels of testosterone and DHT, as well as AR expression in the prostate, in the control group compared to the sham group. These findings are consistent with the results of previous studies [25,44]. TH extract significantly decreased serum testosterone and DHT levels and reduced the protein expression of AR in the prostate. Substantial evidence suggests that higher serum DHT levels and AR expressions are associated with the progression of BPH [45]. Blocking androgen/AR signaling reduces the volume of BPH and alleviates LUTSs [46]. Numerous studies have indicated abundant AR expression in BPH tissue compared to normal prostate tissue, underscoring its pivotal role in BPH progression [47]. Our results suggest that the inhibition of BPH by TH might be attributed to the down-regulation of androgen/AR signaling.

BPH is caused by the proliferation of epithelial and smooth muscle cells of the prostate, predominantly within the transition zone which surrounds the urethra. It results from glandular aging because of the loss of homeostasis, wherein apoptosis plays an important role in maintaining tissue homeostasis [48]. It is known that the disturbance of apoptotic pathways is associated with abnormal cellular proliferation in the prostate [49]. To investigate the potential effects of TH on apoptosis, we assessed the expressions of Bax and Bcl-2 proteins, which are pivotal in the regulation of apoptotic pathways. In the prostate, cells with elevated Bax expression are prone to apoptosis, whereas those with increased expression levels of Bcl-2 are often implicated in hyperplasia, a process marked by the inhibition of apoptosis [50]. Our findings showed that TH reduced Bcl-2 expression and increased that of Bax in the prostate, suggesting that TH promotes the apoptosis of prostate cells, thereby reducing cellular proliferation.

For centuries, Taraxacum spp. (dandelions) have been employed in the treatment of LUTSs and continue to be widely used to this day [51]. Despite this, research on the effectiveness of Taraxacum spp. in treating BPH, a leading cause of LUTSs, remains scarce. Although Lim et al. (2020) [24] demonstrated that a multi-herbal mixture including *T. officinale* exhibits inhibitory effects on BPH by suppressing AR signaling, studies focusing solely on TH are yet to be reported. To the best of our knowledge, the present study is the first to explore the inhibitory effects and potential underlying mechanisms of TH on BPH.

Based on its human safety profile, Taraxacum spp. has long been utilized both as food and as an herbal medicine. The United States Food and Drug Administration designates *T. officinale*, a plant source of TH, as a “generally recognized as safe” substance. The consumption of TH is considered safe and well tolerated in humans when administered in appropriate doses [31]. Moreover, a study demonstrated that the oral administration of TH for four weeks did not adversely affect sperm count or motility in male rats [52]. It is well documented that 5α-reductase inhibitors, such as finasteride, can reversibly impact sperm production and motility negatively [53]. Thus, TH may serve as a safe alternative therapeutic option for BPH.

## 5. Conclusions

This study demonstrated that TH exhibits inhibitory effects on the development of BPH in a TP-induced rat model. These beneficial outcomes may be partially attributed to the regulation of androgen/AR signaling and the induction of apoptosis signaling pathways. Given its recognized safety profile, TH holds promise as a safe and effective alternative treatment for BPH.

## Figures and Tables

**Table 1:** Histoscore.

Variable (Score Values)	Sham	Control	Finasteride	TH 100	TH 300
Low-Power Magnification					
*Luminal shape* regular (1), villous (3), papillary (4), cribriform (5)	1.33 (0.82) ^1^	2.11 (1.05)	1.25 (0.71)	1.89 (1.05)	1.56 (1.13)
*Acinar shape* tubular (1), branched (3), irregular (5)	1.00 (0.00)	2.33 (2.00)	1.00 (0.00)	1.33 (1.00)	1.22 (0.67)
*Interacinar space* large or moderate (1); back-to-back glands (5)	1.00 (0.00)	2.33 (2.00)	1.00 (0.00)	2.67 (2.00)	1.22 (0.67)
*Stroma* fine (1), abundant (3), fibrosis/severe smooth muscle hyperplasia (5)	2.33 (1.03)	2.78 (1.56)	2.50 (1.41)	2.11 (1.45)	2.11 (1.05)
High-Power Magnification					
*Epithelial shape* flattened (1), cuboidal (1), cylindrical (3), hexagonal (5)	1.67 (1.03)	2.33 (1.00)	1.50 (0.93)	1.67 (1.00)	1.22 (0.67)
*Number of layers* mono (1), oligo 2–4 (3), pluri, >5 (5)	1.67 (1.03)	2.56 (0.88)	1.00 (0.00) **	1.67 (1.00)	1.22 (0.67) **
If >1, then add focal (3), diffuse (5)	1.00 (1.55)	2.33 (1.32)	0.00 (0.00) **	1.33 (1.58)	0.33 (1.00) **
*Alignment* polar (1), apolar (3)	1.00 (0.00) *	2.11 (1.05)	1.00 (0.00) *	1.67 (1.00)	1.22 (0.67)
If there is piling up of epithelial cells, add (3)	2.00 (1.55)	3.00 (0.00)	1.50 (1.60)	2.00 (1.50)	1.33 (1.58) *
If there is budding out of epithelial cells into stroma, add (5)	0.00 (0.00) *	3.89 (2.20)	1.88 (2.59)	2.22 (2.64)	2.78 (2.64)
If periacinar clusters of epithelial cells are found, add (3)	0.50 (1.22)	1.00 (1.50)	0.38 (1.06)	1.00 (1.50)	0.00 (0.00)
If isolated clusters of epithelial cells are found outside acini, add (5)	0.83 (2.04) *	3.33 (2.50)	0.00 (0.00) ***	0.00 (0.00) ***	0.56 (1.67) **
*Lesion distribution* (for apolar or budding out cells, no lesion = 0): unilobar: isolated (2): multiple (6). Bilobar: isolated (4); multiple (8)	2.00 (2.19)	5.33 (0.94)	1.50 (2.07) *	3.56 (3.13)	2.67 (3.46)
*Nuclear shape* round, regular (1), irregular (5)	1.00 (0.00) **	3.67 (0.67)	1.00 (0.00) **	2.33 (2.00)	1.89 (1.76)
*Nuclear size* small (2), large (2), small and large in the same acinus (4)	2.00 (0.00) **	3.33 (0.33)	2.00 (0.00) **	2.67 (1.00)	2.22 (0.67) *
*Mitoses per field* absent (0); isolated, 1–2 (2); abundant, 3–5 (5); excessive, >5 (10)	0.33 (0.82) **	2.78 (0.60)	1.00 (1.07) *	0.67 (1.00) **	0.56 (1.67) **
*Basement membrane* intact (1); interrupted (5)	1.00 (0.00) **	4.56 (0.44)	2.00 (1.85) *	2.78 (2.11)	2.33 (2.00) *
Thin (1); thick (5)	1.00 (0.00) **	4.11 (0.59)	1.50 (1.41) **	2.78 (2.11)	1.89 (1.76) *
Total score (arbitrary units)	21.67 (8.36) ***	53.89 (5.23)	22.00 (6.95) ***	34.33 (17.70) *	26.33 (14.31) ***

^1^ Values are presented as the mean with standard deviation in parentheses (*n* = 6 for sham, 8 for finasteride, and 9 for the other groups). * *p* < 0.05, ** *p* < 0.01 and *** *p* < 0.001 vs. control group. Finasteride, finasteride 1 mg/kg; TH 100, Taraxaci Herba extract 100 mg/kg; TH 300, Taraxaci Herba extract 300 mg/kg. Finasteride, finasteride 1 mg/kg.

## Data Availability

The data presented in this study are available on request from the corresponding author due to privacy concerns.

## References

[1] Barry M.J., Fowler F.J., O’Leary M.P., Bruskewitz R.C., Holtgrewe H.L., Mebust W.K., Cockett A.T. (1992). The American Urological Association symptom index for benign prostatic hyperplasia. The Measurement Committee of the American Urological Association. J. Urol..

[2] Egan K.B. (2016). The Epidemiology of Benign Prostatic Hyperplasia Associated with Lower Urinary Tract Symptoms: Prevalence and Incident Rates. Urol. Clin. N. Am..

[3] Madersbacher S., Sampson N., Culig Z. (2019). Pathophysiology of Benign Prostatic Hyperplasia and Benign Prostatic Enlargement: A Mini-Review. Gerontology.

[4] Roehrborn C.G. (2008). Pathology of benign prostatic hyperplasia. Int. J. Impot. Res..

[5] Nicholson T.M., Ricke W.A. (2011). Androgens and estrogens in benign prostatic hyperplasia: Past, present and future. Differentiation.

[6] Vickman R.E., Franco O.E., Moline D.C., Vander Griend D.J., Thumbikat P., Hayward S.W. (2020). The role of the androgen receptor in prostate development and benign prostatic hyperplasia: A review. Asian J. Urol..

[7] Andriole G., Bruchovsky N., Chung L.W., Matsumoto A.M., Rittmaster R., Roehrborn C., Russell D., Tindall D. (2004). Dihydrotestosterone and the prostate: The scientific rationale for 5alpha-reductase inhibitors in the treatment of benign prostatic hyperplasia. J. Urol..

[8] Gao W., Bohl C.E., Dalton J.T. (2005). Chemistry and structural biology of androgen receptor. Chem. Rev..

[9] Sandhu J.S., Bixler B.R., Dahm P., Goueli R., Kirkby E., Stoffel J.T., Wilt T.J. (2024). Management of Lower Urinary Tract Symptoms Attributed to Benign Prostatic Hyperplasia (BPH): AUA Guideline Amendment 2023. J. Urol..

[10] Zlotta A.R., Teillac P., Raynaud J.P., Schulman C.C. (2005). Evaluation of male sexual function in patients with Lower Urinary Tract Symptoms (LUTS) associated with Benign Prostatic Hyperplasia (BPH) treated with a phytotherapeutic agent (Permixon), Tamsulosin or Finasteride. Eur. Urol..

[11] Bullock T.L., Andriole G.L. (2006). Emerging drug therapies for benign prostatic hyperplasia. Expert. Opin. Emerg. Drugs.

[12] McConnell J.D., Bruskewitz R., Walsh P., Andriole G., Lieber M., Holtgrewe H.L., Albertsen P., Roehrborn C.G., Nickel J.C., Wang D.Z. (1998). The effect of finasteride on the risk of acute urinary retention and the need for surgical treatment among men with benign prostatic hyperplasia. Finasteride Long-Term Efficacy and Safety Study Group. N. Engl. J. Med..

[13] Hirshburg J.M., Kelsey P.A., Therrien C.A., Gavino A.C., Reichenberg J.S. (2016). Adverse Effects and Safety of 5-alpha Reductase Inhibitors (Finasteride, Dutasteride): A Systematic Review. J. Clin. Aesthet. Dermatol..

[14] Leisegang K., Jimenez M., Durairajanayagam D., Finelli R., Majzoub A., Henkel R., Agarwal A. (2022). A systematic review of herbal medicine in the clinical treatment of benign prostatic hyperplasia. Phytomed. Plus.

[15] Jang M.H., Ahn T.W. (2015). Inhibitory effects of Taraxacum mongolicum with phreatic water on melanin synthesis. Integr. Med. Res..

[16] Schutz K., Carle R., Schieber A. (2006). Taraxacum—A review on its phytochemical and pharmacological profile. J. Ethnopharmacol..

[17] Di Napoli A., Zucchetti P. (2021). A comprehensive review of the benefits of Taraxacum officinale on human health. Bull. Natl. Res. Cent..

[18] Amritpal Singh A.S., Samir Malhotra S.M., Ravi Subban R.S. (2008). Dandelion (*Taraxacum officinale*)-hepatoprotective herb with therapeutic potential. Pharmacogn. Rev..

[19] Mir M.A., Sawhney S., Jassal M. (2013). Qualitative and quantitative analysis of phytochemicals of Taraxacum officinale. Wudpecker J. Pharm. Pharmocol..

[20] Fan M., Zhang X., Song H., Zhang Y. (2023). Dandelion (*Taraxacum* Genus): A Review of Chemical Constituents and Pharmacological Effects. Molecules.

[21] Lee M.-H., Song S.-H., Ham I.-H., Bu Y.-M., Kim H.-C., Choi H.-Y. (2010). Anti-inflammatory effect and contents from the aerial part and root of the various Taraxacum spp. distributed in Korea. Korea J. Herbol..

[22] Park J.H., Seo B.I. (2011). A philological study on poisoning and side effect of Taraxci Herba. J. Jeahan Orient. Med. Acad..

[23] McVary K.T. (2003). Clinical evaluation of benign prostatic hyperplasia. Rev. Urol..

[24] Lim S., Kim H.K., Lee W., Kim S. (2020). Botanical preparation HX109 inhibits macrophage-mediated activation of prostate epithelial cells through the CCL4-STAT3 pathway: Implication for the mechanism underlying HX109 suppression of prostate hyperplasia. Heliyon.

[25] Choi Y.J., Kim E.K., Fan M., Tang Y., Hwang Y.J., Sung S.H. (2019). Effect of Paecilomyces tenuipes Extract on Testosterone-Induced Benign Prostatic Hyperplasia in Sprague-Dawley Rats. Int. J. Environ. Res. Public Health.

[26] Minh T.D., Thanh Ha T.N., Duy T.N., Hoang N.N., PhamTien D., Thai H.P., Thi H.N., Thi Lan P.D., Quoc B.P., Ivkin D.Y. (2021). Linh Phu Khang Tue Tinh inhibited prostate proliferation in rats induced benign prostatic hyperplasia by testosterone propionate. J. Ethnopharmacol..

[27] Brown C. (2008). Intra-abdominal castration in the rat. Lab. Anim..

[28] Percie du Sert N., Hurst V., Ahluwalia A., Alam S., Avey M.T., Baker M., Browne W.J., Clark A., Cuthill I.C., Dirnagl U. (2020). The ARRIVE guidelines 2.0: Updated guidelines for reporting animal research. BMC Vet. Res..

[29] WHO (1999). WHO Monographs on Selected Medicinal Plants.

[30] Nair A.B., Jacob S. (2016). A simple practice guide for dose conversion between animals and human. J. Basic. Clin. Pharm..

[31] Kania-Dobrowolska M., Baraniak J. (2022). Dandelion (*Taraxacum officinale* L.) as a Source of Biologically Active Compounds Supporting the Therapy of Co-Existing Diseases in Metabolic Syndrome. Foods.

[32] Aremu O.O., Oyedeji A.O., Oyedeji O.O., Nkeh-Chungag B.N., Rusike C.R.S. (2019). In Vitro and In Vivo Antioxidant Properties of Taraxacum officinale in Nomega-Nitro-l-Arginine Methyl Ester (L-NAME)-Induced Hypertensive Rats. Antioxidants.

[33] Scolnik M.D., Servadio C., Abramovici A. (1994). Comparative study of experimentally induced benign and atypical hyperplasia in the ventral prostate of different rat strains. J. Androl..

[34] Golomb E., Kruglikova A., Dvir D., Parnes N., Abramovici A. (1998). Induction of atypical prostatic hyperplasia in rats by sympathomimetic stimulation. Prostate.

[35] Kochakian C.D., Endahl B.R. (1959). Changes in body weight of normal and castrated rats by different doses of testosterone propionate. Proc. Soc. Exp. Biol. Med..

[36] Sorokina I.V., Zhukova N.A., Meshkova Y.V., Baev D.S., Tolstikova T.G., Bakarev M.A., Lushnikova E.L. (2022). Modeling of Benign Prostatic Hyperplasia in Rats with a High Dose of Testosterone. Bull. Exp. Biol. Med..

[37] Zhang J., Zhang M., Tang J., Yin G., Long Z., He L., Zhou C., Luo L., Qi L., Wang L. (2021). Animal models of benign prostatic hyperplasia. Prostate Cancer Prostatic Dis..

[38] Wang Z., Hu L., Salari K., Bechis S.K., Ge R., Wu S., Rassoulian C., Pham J., Wu C.L., Tabatabaei S. (2017). Androgenic to oestrogenic switch in the human adult prostate gland is regulated by epigenetic silencing of steroid 5alpha-reductase 2. J. Pathol..

[39] Pais P. (2010). Potency of a novel saw palmetto ethanol extract, SPET-085, for inhibition of 5alpha-reductase II. Adv. Ther..

[40] Cheng P.-H., Chiu P.-C., Chang J.-C., Lin S.-M., Li Y.-J., Lo D.-Y., Lai L.-R., Wu S.-C., Chiou R.Y.Y. (2021). Inhibition of testosterone-mediated benign prostatic enlargement of orchiectomized Sprague-Dawley rats by diets supplemented with bio-elicited peanut sprout powder (BPSP) and three new BPSP-extracted natural compounds identified. J. Funct. Foods.

[41] Shin I.S., Lee M.Y., Jung D.Y., Seo C.S., Ha H.K., Shin H.K. (2012). Ursolic acid reduces prostate size and dihydrotestosterone level in a rat model of benign prostatic hyperplasia. Food Chem. Toxicol..

[42] Shin S.J., Lee K.H., Chung K.S., Cheon S.Y., An H.J. (2017). The traditional Korean herbal medicine Ga-Gam-Nai-Go-Hyan suppresses testosterone-induced benign prostatic hyperplasia by regulating inflammatory responses and apoptosis. Exp. Ther. Med..

[43] Kemppainen J.A., Lane M.V., Sar M., Wilson E.M. (1992). Androgen receptor phosphorylation, turnover, nuclear transport, and transcriptional activation. Specificity for steroids and antihormones. J. Biol. Chem..

[44] Park H.S., Seo C.S., Wijerathne C.U., Jeong H.Y., Moon O.S., Seo Y.W., Won Y.S., Son H.Y., Lim J.H., Kwun H.J. (2019). Effect of Veratrum maackii on Testosterone Propionate-Induced Benign Prostatic Hyperplasia in Rats. Biol. Pharm. Bull..

[45] Liao C.H., Li H.Y., Chung S.D., Chiang H.S., Yu H.J. (2012). Significant association between serum dihydrotestosterone level and prostate volume among Taiwanese men aged 40–79 years. Aging Male.

[46] Izumi K., Mizokami A., Lin W.J., Lai K.P., Chang C. (2013). Androgen receptor roles in the development of benign prostate hyperplasia. Am. J. Pathol..

[47] Nicholson T.M., Sehgal P.D., Drew S.A., Huang W., Ricke W.A. (2013). Sex steroid receptor expression and localization in benign prostatic hyperplasia varies with tissue compartment. Differentiation.

[48] Torrealba N., Rodriguez-Berriguete G., Vera R., Fraile B., Olmedilla G., Martinez-Onsurbe P., Sanchez-Chapado M., Paniagua R., Royuela M. (2020). Homeostasis: Apoptosis and cell cycle in normal and pathological prostate. Aging Male.

[49] Minutoli L., Rinaldi M., Marini H., Irrera N., Crea G., Lorenzini C., Puzzolo D., Valenti A., Pisani A., Adamo E.B. (2016). Apoptotic Pathways Linked to Endocrine System as Potential Therapeutic Targets for Benign Prostatic Hyperplasia. Int. J. Mol. Sci..

[50] Lee J.Y., Kim S., Kim S., Kim J.H., Bae B.S., Koo G.B., So S.H., Lee J., Lee Y.H. (2022). Effects of red ginseng oil(KGC11o) on testosterone-propionate-induced benign prostatic hyperplasia. J. Ginseng Res..

[51] Hu C. (2018). Taraxacum: Phytochemistry and health benefits. Chin. Herbal. Med..

[52] Mo S. (2019). Effect of increasing nitric oxide and dihydrotestosterone by Taraxacum coreanum extract. J. Appl. Biol. Chem..

[53] Amory J.K., Wang C., Swerdloff R.S., Anawalt B.D., Matsumoto A.M., Bremner W.J., Walker S.E., Haberer L.J., Clark R.V. (2007). The effect of 5alpha-reductase inhibition with dutasteride and finasteride on semen parameters and serum hormones in healthy men. J. Clin. Endocrinol. Metab..

